# Serum paraoxonase and arylesterase activities in patients with lung cancer in a Turkish population

**DOI:** 10.1186/1471-2407-7-48

**Published:** 2007-03-15

**Authors:** Emin T Elkiran, Nefsal Mar, Bilge Aygen, Ferit Gursu, Aziz Karaoglu, Suleyman Koca

**Affiliations:** 1 Department of Internal Medicine, Firat (Euphrates) University School of Medicine, Elazig, Turkey; 2 Department of Biochemistry and Clinical Biochemistry, Firat (Euphrates) University School of Medicine, Elazig, Turkey

## Abstract

**Background:**

Lung cancer (LC) is the leading cause of cancer-related deaths. Oxidative DNA damage may contribute to the cancer risk. The antioxidant paraoxonase (PON1) is an endogenous free radical scavenger in the human body. The aim of this study was to determine serum PON1 and arylesterase (ARE) activities in patients with newly diagnosed LC.

**Methods:**

This case control study involved a total of 39 patients with newly diagnosed LC (untreated) and same number of age- and sex-matched healthy individuals. Serum PON1 and ARE activities in addition to lipid parameters were measured in both groups.

**Results:**

Serum PON1 and ARE activities were found to be lower in patients with LC compared to the controls (p = 0.001 and p = 0.018, respectively). The ratio of PON1/high density lipoprotein (HDL) was significantly lower in the LC group compared to the control one (p = 0.009). There were positive correlations between the serum levels of HDL and PON1 in both the control (r = 0.415, p = 0.009) and the LC groups (r = 0.496, p = 0.001), respectively. PON1 enzyme activity was calculated as three different phenotypes in both groups. In regard to lipid parameters, total cholesterol levels were significantly lower (p = 0.014) in the LC group whereas the other lipid parameters such as HDL, LDL, and triglyceride levels were not significantly different among groups.

**Conclusion:**

Serum PON1 activity is significantly low in the LC group compared with the healthy controls. Metastasis status and cigarette smoking do not affect serum PON1 activity in the LC patients.

## Background

Lung cancer (LC) is among the most common malignancies in the Western World and is the leading cause of cancer deaths in both men and women. It is one of the few tumors with a known carcinogen, namely tobacco, contributing to its etiology. Since cigarette smoking was noted in 80% to 90% of patients with LC, the leading cause of LC is accepted to be smoking [1,2].

An elevated oxidative status has been found in many types of cancer cells, and the introduction of chemical and enzymatic antioxidants can inhibit tumour cell proliferation [3]. High doses and/or inadequate removal of reactive oxygen species (ROS) result in oxidative stress, which may cause severe metabolic malfunctions and damage to biological molecules including DNA [4]. It is well known that oxidative stress induced by environmental carcinogen exposure may affect cellular functions in various pathological conditions, including cancer [5,6].

Human serum paraoxonase (PON1) and arylesterase (ARE) are esterase enzymes that have lipophilic antioxidant characteristics. Serum PON1 binds to high density lipoprotein (HDL) and contributes to the elimination of organophosphorus compounds, such as paraoxon, and carcinogenic lipid soluble radicals from lipid peroxidation. PON1 is one of the endogenous free-radical scavenging systems in the human body [7-9]. Serum PON1 together with ARE have been demonstrated to function as a single enzyme [10]. PON1 activity varies widely among individuals, partly related to polymorphisms. The PON1 gene has two common coding region polymorphisms [11]. A genetic polymorphism of PON1 activity determines high versus low paraoxon hydrolysis in human populations [12]. PON1 also has ARE activity, which does not exhibit activity polymorphism and can therefore serves as an estimate of enzyme protein [13]. Human serum PON1 shows neither age-related change in activity nor gender differences [14]. However, diet, cigarette smoking, acute phase proteins, and pregnancy affect serum PON1 levels and activities [15-17]. Reduced PON1 activities have been reported in several groups of patients with diabetes mellitus, hypercholesterolemia and cardiovascular disease who are under increased oxidative stress [18,19]. There are relatively few studies on in PON1 activity in cancer. Serum levels of PON1 were lower in patients with pancreatic or gastric cancer than in healthy controls in two case-control studies. Despite these initial findings, the interaction of serum PON1 levels with cancer is not completely known [15,20,21]. It has been emphasized that PON1 polymorphisms might contribute to the increased risk of cancer in associated with pollutants and other environmental chemicals [22].

The aim of this study was to investigate the activity of serum PON1 in patients with LC, compared to that of healthy controls, and to investigate possible alterations in relation to the stage and type of LC.

## Methods

### The study population

This case-control study was conducted at the Department of Internal Medicine of the Firat (Euphrates) University Hospital setting in Elazig, Turkey, between December 2004 and June 2005. A total of 44 patients were admitted during the period. However two of them were excluded from the study due to history of taking antilipidemic drug, three of them were also not eligible for the study due to history of taking chemotherapy. The remaining 39 patients with previously untreated, histopathologically verified newly diagnosed as of LC and 39 age- and sex-matched healthy volunteers were enrolled into this study. Informed consents were obtained from patients prior to the study. The study protocol and the procedures were approved by Firat University Local Ethical Committee, and all subjects were recruited upon obtaining informed consent. Subjects' history and physical examinations were documented. The diagnosis of LC was based on histopathologic findings. Nine out of 39 patients were small cell LC, 16 were squamous cell LC, 14 were adenocarcinoma type. Cases either study group or control, who had pathologies that could cause secondary lipid disorders, cardiovascular diseases, diabetes mellitus, renal failure, chronic infection and inflammation, alcohol abuse, and those who used antilipidemic and antioxidant drugs were excluded from the study. Additionally, LC patients with previously performed chemotherapy, radiotherapy and surgery were also excluded from the study.

### Serum samples

Blood samples were collected in tube (BD diagnostics-preanalytic systems, UK) at 0800–0900 A.M. after 8–12 hours of fasting. Professional staff performed venipuncture, using vacutainers to obtain 10 mL of whole blood. Since calcium EDTA inhibits PON1 activity, calcium EDTA containing tubes were not used for collecting serum samples. The samples were centrifuged at 3500 rpm for 5 min. and serum samples were stored at -20°C until assayed.

Serum levels of total cholesterol, triglyceride, HDL-cholesterol and low density lipoprotein (LDL)-cholesterol were determined by means of an Olympus AU 600 autoanalyzer (Olympus Optical Co., Japan) using commercially available assay kits. Serum total cholesterol, triglyceride, HDL-cholesterol and LDL-cholesterol levels were measured at the same day that the blood was collected.

### Assay of serum paraoxonase and arylesterase activities

Paraoxonase activity was determined spectrophotometrically using paraoxon (O, O-diethyl-o-p-nitro-phenylphosphate; Sigma Chemical Co) as the substrate and measured by increases in the absorbance at 412 nm due to the formation of 4-nitrophenol as already described [23]. Briefly, the activity was measured at 25°C by adding 50 μl of serum to 1 ml Tris-HCl buffer (100 nM at pH 8.0) containing 2 mM CaCl2 and 5.5 mM of paraoxon. The rate of generation of 4-nitrophenol was determined at 412 nm with a spectrophotometer (Techcomp 8500 II UV/VIS, China). PON1 activity is expressed in U/l serum. One unit of PON1 activity was defined as 1 nmol of 4-nitrophenol formed per minute under the above assay conditions.

Arylesterase activity was also measured spectrophotometrically using phenylacetate (Sigma Co, London, UK) as the substrate. The phenol formed after the addition of a 40-fold diluted serum sample was measured spectrophotometrically at 217 nm following an established procedure [24]. The activity of ARE was expressed in kU/l serum. One unit was defined as the enzyme quantity that disintegrates 1 nmol phenylacetate per minute.

### The phenotypic distribution of paraoxonase

The phenotypic distribution of PON1 activity was determined by the dual substrate method. Three phenotypes were determined in the study groups by taking the ratio of PON1 activity to arylesterase activity. The ratio of hydrolysis of paraoxon in the presence of 1 M NaCl (salt-stimulated PON1) to the hydrolysis of phenylacetate was used to classify individuals to one of the three possible phenotypes; AA (homozygote low activity), AB (heterozygote activity) and BB (homozygote high activity) [25]. Phenotype of PON1 activity according to the PON1/ARE rate was also performed individually for each group and three different phenotypes were determined for each group. The PON1/ARE rates of LC patients were considered as: 0.56–1.55 AA, 1.56–3.10 AB and 3.11–4.66 BB phenotypes, respectively. The PON1/ARE rates for the control group were considered as: 0.94–1.14 AA, 1.15–2.28 as AB, 2.29–3.50 as BB phenotypes, respectively.

### Statistical analysis

Statistical analyses were performed using the SPSS software package, version 10.0 for Windows. Clinical laboratory data were expressed as mean ± standard deviation. Mean values were compared between patients with LC and healthy individuals by Student's *t *test. Mann Whitney-*U *test was used to assess the mean differences between smoker LC patients and smoker control, and between metastatic and nonmetastatic LC patients. The Pearson's correlation analysis was used to assess the relationship between PON1 activity and HDL cholesterol levels. The analysis of variance (ANOVA) test was used to compare biochemical parameters among small cell carcinoma, squamous cell carcinoma and adenocarcinoma.

Stepwise multivariate linear regression analyses (with 95% confidence intervals) were performed by considering the PON1 activity as the dependent variable. The possible associations between serum PON1 and independent variables (total cholesterol, HDL cholesterol, triglyceride levels, stage of disease and metastasis status) were followed. A value of *p *< 0.05 was considered as statistically significant.

## Results

The demographic features and biochemical parameters of LC patients and controls are presented in the Table 1. Out of the 39 patients with LC, 38 (97%) of the patients were males and 1 (3%) was female. The mean age was 64.4 ± 8.9 years (range, 50–85 years). Thirty had non-small cell LC (16 squamous cell LC, 14 adenocarcinoma type), nine had small cell LC. Patients were staged according to the American Joint Committee on Cancer (AJCC) staging system. Nine patients had stage II disease, 10 had stage III and the remaining 20 had stage IV disease. The control group was consisted of 39 healthy individuals, 38 males and 1 female with mean age of 64.05 ± 8.6 years (range, 50–85 years). The mean age and sex distribution were similar in both groups. In addition, there was no difference in race among groups. The mean serum PON1 activities were 395.8 ± 116.6 U/mL and 252.7 ± 104.4 U/mL in the control group and in the patients with LC, respectively. When compared to healthy controls, serum ARE activity were lower in patients with LC which was statistically significant (Table 1). Serum HDL cholesterol, LDL cholesterol and triglyceride levels were not significantly different among groups. Although not statistically significant, the HDL cholesterol levels, serum HDL level and PON1 activity were lower in LC patients. The PON1 activity was standardized with HDL concentrations (PON1/HDL ratio). It was found that the standardized PON1 enzyme activity was significantly lower in the LC patients (8.1 ± 2.9) as compared with controls (9.8 ± 2.9; p = 0.009). The serum total cholesterol level in patients with LC was significantly lower compared with healthy subjects (p = 0.014) (Table 1).

There was no statistical difference between the biochemical parameters among small cell carcinoma, squamous cell carcinoma and adenocarcinoma in any stage (Table 2).

It was found in the smoker LC patients that serum PON1 and ARE activities was significantly lower compared with smoker controls. Serum PON1 activity in the smoker LC patients was markedly lower (p < 0.001) than that of the smoker control subjects. Similarly, serum ARE activity in the smoker LC patients was lower. Serum ARE activities were 175.3 ± 48.7 U/L and 143.6 ± 57.3 U/L; in the smoker control subjects and in the smoker LC patients, respectively (p = 0.025). In the smoker control subjects, serum HDL cholesterol levels were significantly higher (p < 0.001) than that of the smoker LC patients (Table 3).

As shown in the Table 4, no statistically significant differences were observed in serum PON1 and ARE activities between metastatic and nonmetastatic LC patients. Serum PON1 activity was 259.5 ± 94.9 U/mL in the patients with metastatic LC whereas it was 245.6 ± 115.7 U/mL in the patients with nonmetastatic LC (p = 0.70). The mean serum ARE activities were 153.9 ± 62.9 U/L and 153.9 ± 62.9 U/L in the metastatic LC patients and in the nonmetastatic LC patients (p = 0.09).

We determined the phenotype distribution of our study groups by taking the ratio of PON1 activity to arylesterase activity. The distribution of paraoxonase phenotypes in LC patients and controls was as follows: AA (homozygote low activity), 36% and 3%, AB (heterozygote activity) 51% and 35%, BB (homozygote high activity) 13% and 62%, respectively. Serum PON1 levels in the individuals with AA, AB, and BB phenotypes in the LC group were lower than the same phenotype individuals in the control group.

**Figure 1:**
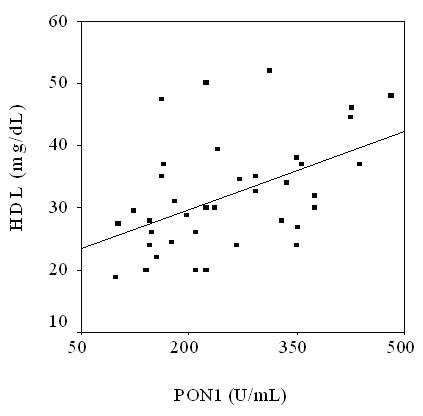
Positive correlation between serum HDL concentration and PON1 activity in the LC group (r = 0.496, p = 0.001).

**Figure 2:**
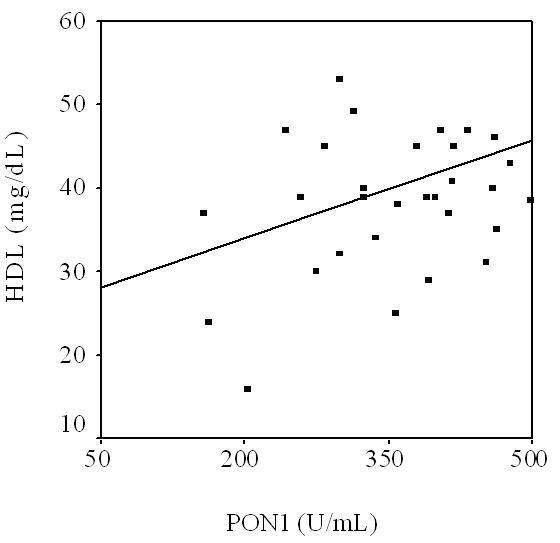
Positive correlation between serum HDL concentration and PON1 activity in the control group (r = 0.415, p = 0.009).

There was established positive correlation between serum PON1 activity and serum level of HDL cholesterol (r = 0.496, p = 0.001) in patients with LC. Similarly, serum PON1 activity also correlated positively with serum HDL cholesterol level (r = 0.415, p = 0.009) in the control group (Figures 1 and 2).

Multiple linear regression analysis of the variables was also performed by considering the PON1 as the dependent variable. It was determined that total cholesterol, HDL cholesterol, triglyceride levels, stage of disease and presence or absence of metastasis was not the determining factor for the PON1 activity (Table 5).

## Discussion

Reactive oxygen species molecules are highly reactive and can attack almost every cell component, causing further damage to the surrounding tissues. An elevated oxidative stress and free oxygen radicals have been associated with the increased risks of various cancers. End products of lipid peroxidation and scavenging system elements have been thought to play a role in oncogenesis [26-28]. Kaynar et al. studied the activities of antioxidant enzymes and demonstrated that erythrocyte malondialdehyde, nitric oxide, total glutathione levels and erythrocyte superoxide dismutase, catalase and xanthine oxidase activities were significantly higher in patients with LC than in controls. This study indicates significant changes in antioxidant defense system in LC patients, which may lead to enhanced action of oxygen radicals, resulting in lipid peroxidation [29]. One of the lipid peroxidation end products, malondialdehyde level in patients with LC has been found significantly higher than those in controls [30]. Carcinogenic lipid soluble radicals are formed as a result of lipid peroxidation and PON1 binds to these radicals. PON1 has been shown to metabolize lipid-soluble radicals [15,31]. Serum PON1 activity was suggested to be inversely associated with oxidative stress in serum and macrophages and that PON1 deficiency results in increased oxidative stress [32]. In our study, serum PON1 activity was found to be significantly lower in patients with LC compared to healthy individuals. Some studies have established a positive correlation between PON1 mutations and the risk of cancer. However, the data are conflicting and other studies revealed no evidence for an association with malignancy. A case-control study which contains 177 patients by Lee et al. [33] showed that the risk of developing LC were significantly increased in individuals carrying the PON1 gene Q/Q genotype. It was also stated that, this correlation could not be established with the R/R or Q/R genotypes of the PON1 gene in this study. To the best of our knowledge, our study is the first to state the association between PON1 and LC in the English Literature. Similar to the aforementioned study, we observed that in LC patients, PON1 activity was reduced and not influenced by the cigarette smoking and metastasis status of subjects. In our study, we have not performed any genotypical analysis due to technical limitations. Kerridge et al. [34] found that PON1 BB gene (Arg 192 isoform) polymorphism was associated with risk of non-Hodgkin's lymphoma. A cohort study found no association between PON1 polymorphism and prostate cancer [35], whereas another case-control study demonstrated that men with PON192/QQ had a significantly increased risk for prostate cancer compared with PON192/RR genotype [36]. In a prospective study of Stevens et al. it was reported that L55 M PON1 genotype may have increased risk factor for breast cancer in postmenopausal women, whereas the Q192R single nucleotide polymorphism was not associated with breast cancer incidence [37]. The study of Kafadar et al. showed that serum PON1 activity is significantly lower in patients with high grade glioma and meningioma compared to control subjects [38]. Other investigators have not reported any significant differences in PON1 genotype distributions in patients with colorectal cancer comparison to healthy individuals [39].

In our study, we found decreased activities of PON1 and ARE enzymes in patients with LC comparison to the group of healthy individuals. To assess whether the observed reduction of PON1 activity was due to the reduced HDL level, we standardized the enzyme activity for HDL concentration (PON1/HDL) and found that the standardized enzyme activities were lower in patient group compared to the controls (p = 0.009). Positive correlation was determined between serum PON1 activity and HDL cholesterol. These data indicate that PON1 activity changes are not entirely dependent on HDL concentration in this study group.

Lipid peroxidation, as a well-known index of ROS activity, is an oxidative stress associated with membrane lipid destruction. Previous studies have reported increased lipid peroxidation in cancer tissues, in serum and in erythrocytes of patients with LC [30,40,41]. The serum PON1 activity is highly variable and its regulation is complex. Moreover the mechanism of the reduction of serum PON1 activity in LC patients is not clearly understood. This reduction could be related to increased lipid peroxidation, since oxidized lipids are reported to inhibit PON1 activity [42]. In addition, the activity of PON1 has been reported to be significantly reduced in some conditions accompanying oxidative stress and inflammatory conditions, including rheumatoid arthritis, ulcerative colitis and Behcet's disease [43,44]. Similarly, reduced serum PON1 activity in patients with LC could be a result of increased activity of ROS in LC. Cigarette smoking has also been shown to decrease serum PON1 levels and activity [45]. In our study, when smoking status of each group were considered, we found that in the smoker LC patients, serum PON1 and ARE activities was significantly lower compared with smoker healthy controls (p < 0.001). Standardized enzyme activities of PON1/HDL were also lower in smoker patient group compared to smoker controls (p = 0.047). These results suggest the reduction of PON1 activity in patients with LC was independent from cigarette smoking.

## Conclusion

This study showed that serum PON1 and ARE activities were significantly lower in LC patients compared to healthy subjects. These observations suggested the hypothesis that defects in the antioxidant system capacity and altered PON1 activity may be involved in the pathogenesis of LC. But, these preliminary results are needed to be verified by large numbered prospective studies to reveal the possible association between PON1 and LC.

## Competing interests

The author(s) declare that they have no competing interests.

## Authors' contributions

ETE: Have made substantial contributions to conception, design, interpretation of data, and drafting the manuscript.

NM: Have made substantial contributions to acquisition of data and design of the study.

BA: Have been involved in drafting the manuscript and contributed to the intellectual content of the manuscript and design of the study.

FG: Have been involved in acquisition, analysis and interpretation of the data.

AK: Have contributed to the intellectual content of the manuscript.

SK: Have been involved in acquisition, statistical analysis of the data.

All authors read and approved the final manuscript.

## Pre-publication history

The pre-publication history for this paper can be accessed here:



## Figures and Tables

**Table 1:** Demographic features, paraoxonase activity and biochemical parameters in controls and lung cancer patient groups

	Control group (n = 39)	Patient group (n = 39)	*P *value
Age (year)	64.0 ± 8.6	64.4 ± 8.9	0.861
Cigarette smoking (%)	64.1	89.7	0.014
Total cholesterol (mg/dl)	193.2 ± 48.5	165.1 ± 50	0.014
LDL cholesterol (mg/dl)	128.8 ± 35.3	116 ± 42.4	0.152
HDL cholesterol (mg/dl)	41.7 ± 10.9	34.9 ± 20.8	0.074
VLDL cholesterol (mg/dl)	32 ± 16.3	29.3 ± 11.5	0.393
Triglyceride (mg/dl)	160.8 ± 81.7	141.3 ± 53.9	0.219
PON1 (U/mL)	395.8 ± 116.6	252.7 ± 104.4	0.001
ARE (U/L)	167.7 ± 45	137.4 ± 57.6	0.018
PON1/ARE	2.4 ± 0.6	2.0 ± 0.9	0.008
PON/HDL	9.8 ± 2.9	8.1 ± 2.9	0.009

Values are mean ± S.D. PON1: paraoxonase, ARE: arylesterase

**Table 2:** Serum paraoxonase activity and biochemical parameters in small cell lung cancer adenocarcinoma and epidermoid carcinoma patient groups

	SCLC (n = 9)	AC (n = 14)	EC (n = 16)	*P *value
Total cholesterol (mg/dl)	158.9 ± 30.7	170.9 ± 45.6	163.5 ± 63.1	0.849
LDL cholesterol (mg/dl)	115.4 ± 26.3	118.2 ± 41.1	114.4 ± 52.1	0.971
HDL cholesterol (mg/dl)	30.3 ± 4.3	34.2 ± 9.9	38 ± 31.3	0.680
VLDL cholesterol (mg/dl)	33.9 ± 15	27.8 ± 8.7	28.1 ± 11.6	0.504
Triglyceride (mg/dl)	158.8 ± 68.8	131.4 ± 37.1	140.1 ± 58.2	0.395
PON1 (U/mL)	233.9 ± 70.5	273.1 ± 118.1	245.4 ± 110.5	0.649
ARE (U/L)	163 ± 69.5	115.7 ± 44.2	142 ± 57.2	0.144
PON1/ARE	1.7 ± 0.9	2.5 ± 0.9	1.8 ± 0.7	0.062

Values are mean ± S.D. SCLC:Small Cell carcinoma, AC: Adenocarcinoma, EC:Epidermoid carcinoma, PON1: paraoxonase, ARE: arylesterase

**Table 3:** Paraoxonase and arylesteraz activies in smoker controls and smoker lung cancer patients

	Smoker controls (n = 25)	Smoker LC patients (n = 35)	*P *value
PON1 (U/mL)	412.9 ± 121.9	263.8 ± 104.5	0.001
ARE (U/L)	175.3 ± 48.7	143.6 ± 57.3	0.025
HDL cholesterol (mg/dl)	42.6 ± 11.1	32.6 ± 8.6	0.001
PON1/HDL cholesterol	10 ± 3.2	8.3 ± 3.3	0.047

Values are mean ± S.D. LC: Lung cancer, PON1: paraoxonase, ARE: arylesterase

**Table 4:** Paraoxonase activity and biochemical parameters in metastatic and non-metastatic lung cancer patients

	Nonmetastatic LC patients (n=19)	Metastatic LC patients (n=20)	*P *value
Total cholesterol (mg/dl)	169.7 ± 57.6	160.7 ± 42.7	0.66
LDL cholesterol (mg/dl)	115.3 ± 45.8	116.6 ± 40.1	0.94
HDL cholesterol (mg/dl)	38.8 ± 28.5	31.1 ± 8.1	0.62
Triglyceride (mg/dl)	149.9 ± 55.7	133.2 ± 52.4	0.41
PON1 (U/mL)	245.6 ± 115.7	259.5 ± 94.9	0.70
ARE (U/L)	120 ± 47	153.9 ± 62.9	0.09

Values are mean ± S.D. LC: Lung cancer, PON1: paraoxonase, ARE: arylesterase

**Table 5:** Multiple linear regression model with paraoxonase as the dependent variable

	Standardized Coefficients Beta	t	*P*	95% CI
95% CI				
Age	-0.050	-0.650	0.521	-2.4 to 1.3
Sex	0.028	0.356	0.725	-85.9 to 121.9
Total cholesterol	0.414	1.754	0.092	-0.2 to 1.9
LDL cholesterol	-0.260	-1.115	0.276	-1.8 to 0.5
HDL cholesterol	-0.118	-1.378	0.181	-1.5 to 0.3
VLDL cholesterol	-0.294	-1.415	0.169	-1.4 to 0.3
Triglyceride	0.089	0.447	0.659	-2.9 to 4.5
Stage	0.248	1.146	0.263	-26.7 to 93.7
Metastasis status	-0.266	-1.258	0.220	-144. to 34.9

CI: Confidence Interval
